# Cross-Sectional Study to Compare Sentinel Lymph Node Biopsy vs. Complete Axillary Lymph Node Dissection for the Evaluation of Lymph Node Metastasis in Females With Locally Advanced Breast Carcinoma Post Neoadjuvant Chemotherapy

**DOI:** 10.7759/cureus.107344

**Published:** 2026-04-19

**Authors:** Rahul Patel, Fareed Khan, Abhay K Brahamane, Oshin Chhipa

**Affiliations:** 1 General Surgery, Mahatma Gandhi Memorial Medical College and Maharaja Yeshwantrao (MY) Hospital, Indore, IND

**Keywords:** alnd, axillary lymph node dissection, breast carcinoma, labc, sentinel lymph node biopsy, slnb, surgical de-escalation

## Abstract

Background

Accurate axillary staging after neoadjuvant chemotherapy (NACT) in locally advanced breast cancer is essential for treatment planning. Sentinel lymph node biopsy (SLNB) offers a less morbid alternative to complete axillary lymph node dissection (CALND), but its diagnostic performance after NACT requires evaluation.

Aim

To evaluate the diagnostic accuracy, specifically the sensitivity and false-negative rate, of SLNB compared to CALND in identifying residual axillary metastasis among females with locally advanced breast carcinoma who achieve a clinically node-negative (cN0) status following neoadjuvant chemotherapy in a tertiary care setting.

Materials and methods

This prospective, cross-sectional, hospital-based observational study was conducted over one year at a tertiary care teaching hospital in central India. Forty-five consenting females (≥18 years) with cytologically or histologically proven locally advanced breast cancer, who were clinically node-negative after NACT, underwent intraoperative dye-guided SLNB with lower axillary sampling followed by completion axillary lymph node dissection. Histopathology from SLNB and axillary lymph node dissection (ALND) specimens was compared. Diagnostic indices (sensitivity, specificity, predictive values, and accuracy) were calculated using ALND as the reference standard.

Results

Among 45 subjects, 64.44% were younger than 50 years, and tumors were more often left-sided (60.0%). Invasive ductal carcinoma (IDC) was the most common histologic type (64.4%), and no residual tumor (NRT) was reported in 24.4% of patients. Mean tumor size was 2.15 ± 1.00 cm. SLNB was positive in 18 cases and negative in 27 cases; remaining axillary nodes were positive in 11 and negative in 34 subjects. SLNB demonstrated a sensitivity of 88.89%, specificity of 72.22%, false-negative rate of 11.11%, false-positive rate of 27.77%, positive predictive value of 44.44%, negative predictive value of 96.30%, and overall accuracy of 75.56%.

Conclusion

SLNB showed high sensitivity and a very high negative predictive value as compared with CALND for post-NACT axillary evaluation in clinically node-negative locally advanced breast cancer. The present findings support SLNB as a preliminary and hypothesis-generating alternative to CALND in the post-NACT setting for clinically node-negative LABC in resource-limited settings, while emphasizing that careful patient selection and adherence to best-practice technical standards are essential to maintain diagnostic reliability.

## Introduction

Breast cancer is the most frequently diagnosed malignancy in women worldwide and remains a leading cause of cancer-related mortality, highlighting the ongoing need to refine staging and treatment pathways that preserve oncologic outcomes while reducing treatment-related morbidity [1].

In locally advanced breast carcinoma (LABC), neoadjuvant chemotherapy (NACT) is widely used to downstage the primary tumor and axilla, facilitate operability, and increase the likelihood of achieving a pathological complete response (pCR) [2]. pCR after neoadjuvant treatment is clinically important because it is associated with improved long-term outcomes across breast cancer subtypes and is increasingly used as an indicator of treatment effectiveness in neoadjuvant strategies [3].

Assessment of axillary nodal status remains central to prognosis and to guiding locoregional and systemic treatment decisions after NACT. Complete axillary lymph node dissection (CALND) has historically served as a definitive method to evaluate residual nodal disease; however, it is associated with substantial morbidity, particularly arm lymphedema and functional impairment, which can adversely impact quality of life [4].

SLNB offers a less invasive alternative that aims to stage the axilla with reduced morbidity, but its accuracy following NACT, especially in patients with initially involved nodes, has been an area of active evaluation. Prospective data demonstrate that identification rates and false-negative rates after NACT can vary, depending on patient selection and technical factors, supporting careful assessment of SLNB performance in this setting [5,6]. Systematic reviews and meta-analyses further suggest that SLNB after NACT can be feasible and reliable in biopsy-proven node-positive patients but emphasize the importance of optimized techniques and appropriate interpretation of diagnostic performance measures [5-7].

Therefore, the present study was undertaken to compare the efficacy of SLNB versus CALND in evaluating lymph node metastasis in females with locally advanced breast carcinoma after NACT in the resource-limited setting of a tertiary care centre in central India.

## Materials and methods

Study design and setting

This was a prospective, cross-sectional, hospital-based observational study conducted in the Department of General Surgery, M.G.M. Memorial Medical College and M.Y. Hospital (including associated hospitals), Indore, Madhya Pradesh, India. The study duration was one year from the date of approval by the Institutional Ethics Committee. The study population comprised female patients with LABC reporting to the study center for surgical management after receiving NACT. The institutional protocol implemented the TCH (Trastuzumab (Herceptin), Carboplatin, and Docetaxel (Taxotere), 21 day regimen, 4-6 cycles, followed by Trastuzumab continued for 1 year) chemotherapy protocol; the 4AC + 4T (four cycles of Adriamycin (doxorubicin) and Cytoxan (cyclophosphamide), followed by four cycles of Taxol (paclitaxel), each cycle given every two weeks) chemotherapy protocol for ER+ve (estrogen receptor positive molecular subtype) and other molecular subtypes. Appropriate neo-adjuvant hormonal therapy was given, subject to the availability of hormone receptor status, which was not routinely performed in the institute due to resource constraints. A total of 45 participants were prospectively enrolled using a consecutive sampling approach to populate the study cohort.

Participants and eligibility

All consenting females aged ≥18 years with histologically proven LABC undergoing total mastectomy with axillary lymph node dissection (ALND) after NACT were included. Patients were recruited from those presenting to the Department of General Surgery for modified radical mastectomy (MRM) following NACT. Preoperative axillary assessment was undertaken by systematic clinical evaluation. Exclusion criteria included patients unfit for surgery, patients lost to follow-up, patients undergoing simple mastectomy, and patients with operable breast cancer with clinically positive axilla. Written informed consent was obtained from all participants prior to enrollment, and confidentiality of patient information was maintained throughout the study.

Sentinel lymph node biopsy and axillary procedures

All enrolled patients (n = 45) who were clinically node-negative after NACT underwent SLNB along with completion ALND during the same operative setting. SLNB was performed using a traditional dye injection technique (methylene blue). The dye was injected intraoperatively near the tumor, in the subareolar region, and the axilla was explored after a waiting period of 10-20 minutes to allow lymphatic migration. Sentinel nodes were identified visually as blue-stained nodes; additionally, any immediately adjacent enlarged palpable nodes were removed as part of SLNB with lower axillary sampling (SLNB + LAS). The anatomical boundaries of lower axillary dissection (LAS) were defined as follows: anteriorly by the undersurface of pectoralis major, posteriorly by latissimus dorsi, medially by the second digitation of serratus anterior, superiorly by the intercostobrachial nerve, laterally by the pectoralis minor, and inferiorly by the lower border of the pectoralis minor, including the axillary tail. All patients then underwent completion ALND, performed through a 3-4 cm axillary incision. The boundaries of ALND were defined anteriorly by the pectoralis major and minor muscles and the clavipectoral fascia; posteriorly by the subscapularis, scapula, teres major, and latissimus dorsi; laterally by the thoracodorsal pedicle; medially by the Halsted (costoclavicular) ligament; superiorly by the axillary vein (apex); and inferiorly by the angular vein. The SLNB/LAS specimen and ALND specimen were sent separately for histopathological examination.

Histopathology and outcome measures

The primary study variable was the histopathological status of lymph node metastasis in the SLNB/LAS specimen compared with the remaining axillary nodes from ALND. SLNB performance was assessed by comparison against ALND as the reference standard.

The lymph nodes were fixed in 10% neutral, buffered formalin and macroscopically categorized as either grossly positive or grossly negative. The nodes were then processed, embedded in paraffin, and sectioned at 2 mm intervals. Sections were stained with Hematoxylin and Eosin (H&E) and examined for metastatic deposits. No additional H&E levels or immunohistochemistry (IHC) studies were performed to detect metastasis.

Data were recorded prospectively and analyzed at the end of the study period.

Statistical analysis

Statistical analysis was performed using SPSS version 21 (IBM Corporation, Chicago, IL, USA). Descriptive statistics were used to summarize data as frequencies, percentages, and measures of central tendency and dispersion (mean ± standard deviation). Inferential testing included the independent t-test where applicable. A p-value <0.05 was considered statistically significant, and results were interpreted at the 95% confidence level.

Ethical considerations

Ethical clearance was obtained from the Institutional Ethics Committee prior to the commencement of the study. Written informed consent was obtained from all participants. Patient identities were kept confidential, and the study utilized materials routinely available in the hospital to avoid any additional financial burden to participants.

## Results

A total of 45 study subjects were included (Table 1). The most represented age group was 41-50 years (21/45; 46.7%), followed by 31-40 years and 61-70 years (7/45 each; 15.6%). Participants aged >70 years accounted for 11.1% (5/45), while those aged 51-60 years comprised 8.9% (4/45). Only one participant was aged 21-30 years (2.2%). When dichotomized by age, 64.44% (29/45) were <50 years and 35.56% (16/45) were ≥50 years. Tumors were more frequently located in the left breast (27/45; 60.0%) than in the right breast (18/45; 40.0%) (Table 1).

**Table 1:** Demographic characteristics and tumor laterality of study subjects (n = 45)

Variable	Category	Frequency	Percent (%)
Age group (years)	21–30	1	2.2
	31–40	7	15.6
	41–50	21	46.7
	51–60	4	8.9
	61–70	7	15.6
	>70	5	11.1
Age category	<50	29	64.44
	>50	16	35.56
Tumor laterality	Left breast	27	60.0
	Right breast	18	40.0

Histopathological tumor type distribution is presented in Table 2. Invasive ductal carcinoma (IDC) was the predominant tumor type, observed in 64.4% (29/45) of subjects. No residual tumor (NRT) was reported in 24.4% (11/45). Other tumor types were infrequent: infiltrating lobular carcinoma (ILC) occurred in 4.4% (2/45), while ductal carcinoma in situ (DCIS), IDC with mucinous features, and mucinous carcinoma were each identified in 2.2% (1/45) of cases (Table 2).

**Table 2:** Tumor type distribution among study subjects (n = 45) DCIS: Ductal Carcinoma In Situ, IDC: Infiltrating Ductal Carcinoma, ILC: Infiltrating Lobular Carcinoma; NRT: No Residual Tumor

Tumor type	Frequency	Percent (%)
DCIS	1	2.2
IDC	29	64.4
IDC with mucinous features	1	2.2
ILC	2	4.4
Mucinous carcinoma	1	2.2
NRT	11	24.4
Total	45	100.0

Tumor grade distribution and tumor size characteristics are summarized in Table 3. Grade 3 tumors were most common (37.77%; 17/45), closely followed by grade 2 tumors (35.55%; 16/45). Grade 1 tumors were rare (2.22%; 1/45). NRT was documented in 24.44% (11/45) of cases (Table 3). Tumor size ranged from 0.20 cm to 4.00 cm, with a mean ± SD of 2.15 ± 1.00 cm (Table 3).

**Table 3:** Tumor grade and tumor size characteristics (n = 45) SD: Standard Deviation

Variable	Category / Statistic	Value
Tumor grade	Grade 1	1 (2.22%)
	Grade 2	16 (35.55%)
	Grade 3	17 (37.77%)
	No residual tumor (NRT)	11 (24.44%)
Tumor size (cm)	Minimum	0.20
	Maximum	4.00
	Mean ± SD	2.15 ± 1.00

The comparison between SLNB and the remaining axillary lymph nodes is shown in Table 4. SLNB was positive in 18 subjects, of whom 8 had positive remaining axillary lymph nodes and 10 had negative remaining nodes. SLNB was negative in 27 subjects, with 1 showing positive remaining nodes and 26 showing negative remaining nodes. Overall, the remaining axillary lymph nodes were positive in 9 subjects and negative in 36 subjects (Table 4).

**Table 4:** Sentinel lymph node biopsy (SLNB) validated against the remaining axillary lymph nodes with percentages in parentheses (n = 45) SLNB: Sentinel Lymph Node Biopsy; LN: Lymph Node

SLNB result	Remaining LN Positive	Remaining LN Negative	Total
Positive	8 (17.77%)	10 (22.22%)	18 (40%)
Negative	1 (2.22%)	26 (57.77%)	27 (60%)
Total	9 (20%)	36 (80%)	45 (100%)

Diagnostic performance indices for SLNB, using axillary lymph node dissection as the reference standard, are reported in Table 5. SLNB demonstrated a sensitivity of 88.89% (95% CI: 51.75-99.72) and a specificity of 72.22% (95% CI: 54.81-85.80). The false-negative rate was 11.11% (95% CI: 0.30-48.10), and the false-positive rate was 27.77% (95% CI: 9.58-34.60). The positive predictive value was 44.44% (95% CI: 31.04-58.71), while the negative predictive value was 96.30% (95% CI: 80.20-99.40). Overall accuracy was 75.56% (95% CI: 60.46-87.12) (Table 5).

**Table 5:** Diagnostic performance of sentinel lymph node biopsy (SLNB) compared with complete axillary lymph node dissection (CALND) (gold standard)

Parameter	Value (%)	95% Confidence Interval
Sensitivity	88.89	51.75 – 99.72
Specificity	72.22	54.81 – 85.80
False negative rate	11.11	0.30 – 48.10
False positive rate	27.77	9.58 – 34.60
Positive predictive value	44.44	31.04 – 58.71
Negative predictive value	96.30	80.20 – 99.40
Accuracy	75.56	60.46 – 87.12

## Discussion

The present cohort (n = 45) was dominated by patients younger than 50 years (64.44%), with the highest representation in the 41-50-year group (46.7%). This age profile is clinically relevant because axillary staging decisions increasingly aim to balance oncologic safety with minimization of treatment-related morbidity, particularly in patients likely to receive multimodality therapy. Tumor laterality showed a modest left-sided predominance (60.0%), which is routinely reported for complete clinicopathologic description but is not expected to directly influence axillary staging performance.

IDC was the most frequent histologic subtype (64.4%), consistent with distributions typically reported in breast cancer series. A substantial proportion of cases were categorized as NRT (24.4% by tumor type and grade), indicating that a notable subset had no residual disease on pathology. This finding highlights the effectiveness of neoadjuvant treatment in achieving pathologic clearance in a fraction of patients. Accurate reporting of residual disease status is crucial in this setting, as it directly influences the observed prevalence of nodal metastasis and, consequently, impacts diagnostic performance metrics such as predictive values.

Tumor grade distribution showed that grade 3 (37.77%) and grade 2 (35.55%) tumors were most common, whereas grade 1 tumors were rare (2.22%). Tumor size ranged from 0.20 to 4.00 cm (mean ± SD, 2.15 ± 1.00 cm), reflecting heterogeneity in tumor burden that may contribute to variability in axillary involvement. Together, these clinicopathologic features provide essential context for interpreting SLNB accuracy in this cohort.

When SLNB results were compared with the remaining axillary lymph nodes, discordance was observed in both directions: one false-negative case (SLNB negative with remaining nodes positive) and multiple cases in which SLNB was positive while the remaining nodes were negative. The diagnostic profile in this cohort demonstrated high sensitivity (88.89%) and a very high negative predictive value (96.30%), supporting the clinical usefulness of a negative SLNB result for excluding additional axillary disease. These findings align with large prospective validation studies demonstrating that SLNB can accurately stage the axilla in clinically node-negative breast cancer with acceptable false-negative performance when performed using standard techniques. Krag et al. reported technical outcomes that supported SLNB as a reliable staging approach, providing foundational evidence for its widespread adoption [8].

A key rationale for preferring SLNB over routine ALND is reduced morbidity. Ashikaga et al. demonstrated that patients undergoing SLNB alone experienced less postoperative morbidity than those undergoing ALND, supporting practice patterns that avoid ALND when it is not required for regional control or treatment planning [9]. In the current cohort, the high negative predictive value similarly supports the role of SLNB as a morbidity-sparing strategy when negative, although confidence intervals were wide, reflecting the modest sample size.

The management implications of SLNB findings should also be interpreted in the context of contemporary axillary de-escalation evidence. In selected patients with limited sentinel node metastasis, omission of completion ALND did not compromise outcomes in ACOSOG Z0011, as reported by Giuliano et al. and confirmed with long-term follow-up by Giuliano et al. [10,11]. Likewise, Galimberti et al. [12] showed that ALND could be avoided in patients with sentinel-node micrometastases without loss of disease control, and Donker et al. [13] demonstrated that axillary radiotherapy can provide comparable regional control to ALND after a positive sentinel node, with less morbidity. These trials collectively emphasize that a positive SLNB does not uniformly mandate ALND and highlight the importance of integrating SLNB results with tumor burden, planned breast treatment, and systemic therapy.

In this cohort, specificity (72.22%) and positive predictive value (44.44%) were lower than sensitivity and negative predictive value. This pattern should be interpreted cautiously because predictive values are prevalence-dependent and can be affected by how the reference standard is defined. Additionally, classifying SLNB-positive/remaining-node-negative cases as "false positives" reflects the chosen comparison against remaining axillary nodes; clinically, metastasis confined to the sentinel node is biologically plausible and does not necessarily represent an SLNB error. From a guideline perspective, current recommendations emphasize evidence-based application of SLNB and selective omission or tailoring of further axillary treatment in appropriate early-stage settings, as summarized in the updated ASCO guidelines by Park et al. [14].

SLNB performance can vary depending on initial nodal status and technical factors. Boughey et al. reported that SLNB after neoadjuvant chemotherapy in initially node-positive patients can have higher false-negative rates unless optimized approaches are used (for example, adequate sentinel node retrieval and standardized mapping techniques) [15]. Thus, we can use additional standardised approaches to improve the performance further, as demonstrated by various studies [5-7].

Limitations

This study has certain limitations that merit consideration. First, the relatively small sample size of 45 participants may limit the statistical power of the findings and reduce their representativeness of the wider population. Second, the single-center design introduces the potential for institutional bias, as variations in clinical protocols, patient demographics, and surgeon expertise could have influenced the observed outcomes, thereby restricting the generalizability of the conclusions to other clinical settings. Additionally, variability in tumor characteristics and individual responses to neoadjuvant chemotherapy may have impacted the accuracy of sentinel lymph node biopsy (SLNB) assessments. Furthermore, because diagnostic accuracy varies markedly across SLNB methodologies (dye-only vs dual mapping), this could have led to a misappraisal of the SLNB results in this study. The lack of documented pre‑NACT tumor size constrains the precise evaluation of the chemotherapy‑induced reduction in tumor burden. The absence of hormone receptor status data constrains the accuracy of interpretation and simultaneously underscores a deficiency in the standard treatment pathway, emphasizing the need for corrective measures within resource‑limited healthcare settings.

Overall, the present findings support SLNB as a preliminary and hypothesis-generating alternative to CALND in the post-NACT setting for clinically node-negative LABC in resource-limited settings while emphasizing that careful patient selection and adherence to best-practice technical standards are essential to maintain diagnostic reliability.

## Conclusions

In this tertiary care center cohort of women with locally advanced breast cancer (LABC) treated with neoadjuvant chemotherapy (NACT) in a resource-limited setting, sentinel lymph node biopsy (SLNB) using a dye-only technique demonstrated high sensitivity with a wide confidence interval and a very high negative predictive value (NPV) compared with completion axillary lymph node dissection (ALND). The observed false-negative rate is 11.11%. However, the relatively small sample size and the wide confidence intervals limit the precision of these estimates. These findings should therefore be interpreted with caution and considered preliminary and hypothesis‑generating rather than confirmatory. While the high NPV supports the potential role of SLNB in minimizing unnecessary axillary surgery in clinically node‑negative patients after NACT, the technique’s reliability remains uncertain in this setting. Larger, adequately powered studies with standardized procedural protocols and comprehensive reporting are warranted to validate these results and guide evidence‑based axillary de‑escalation strategies.
